# Dynamic changes in cell size and corresponding cell fate after optic nerve injury

**DOI:** 10.1038/s41598-020-77760-1

**Published:** 2020-12-10

**Authors:** Benjamin M. Davis, Li Guo, Nivedita Ravindran, Ehtesham Shamsher, Veerle Baekelandt, Hannah Mitchell, Anil A. Bharath, Lies De Groef, M. Francesca Cordeiro

**Affiliations:** 1 Institute of Ophthalmology, University College London, 11-43 Bath Street, London, EC1V 9EL UK; 2 Western Eye Hospital, ICORG, Imperial College London, London, NW1 5QH UK; 3 Neural Circuit Development and Regeneration Research Group, Department of Biology, KU Leuven, Naamsestraat 61, 3000 Leuven, Belgium; 4 Laboratory for Neurobiology and Gene Therapy, Department of Neurosciences, KU Leuven, Kapucijnenvoer 33, 3000 Leuven, Belgium; 5 School of Mathematics and Physics, Queens University Belfast, Belfast, Ireland; 6 Department of Bioengineering, Imperial College London, South Kensington Campus, London, UK

**Keywords:** Computational biology and bioinformatics, Image processing, Cell death in the nervous system, Cell death, Retinal diseases

## Abstract

Identifying disease-specific patterns of retinal cell loss in pathological conditions has been highlighted by the emergence of techniques such as Detection of Apoptotic Retinal Cells and Adaptive Optics confocal Scanning Laser Ophthalmoscopy which have enabled single-cell visualisation in vivo. Cell size has previously been used to stratify Retinal Ganglion Cell (RGC) populations in histological samples of optic neuropathies, and early work in this field suggested that larger RGCs are more susceptible to early loss than smaller RGCs. More recently, however, it has been proposed that RGC soma and axon size may be dynamic and change in response to injury. To address this unresolved controversy, we applied recent advances in maximising information extraction from RGC populations in retinal whole mounts to evaluate the changes in RGC size distribution over time, using three well-established rodent models of optic nerve injury. In contrast to previous studies based on sampling approaches, we examined the whole Brn3a-positive RGC population at multiple time points over the natural history of these models. The morphology of over 4 million RGCs was thus assessed to glean novel insights from this dataset. RGC subpopulations were found to both increase and decrease in size over time, supporting the notion that RGC cell size is dynamic in response to injury. However, this study presents compelling evidence that smaller RGCs are lost more rapidly than larger RGCs despite the dynamism. Finally, using a bootstrap approach, the data strongly suggests that disease-associated changes in RGC spatial distribution and morphology could have potential as novel diagnostic indicators.

## Introduction

Glaucoma comprises a distinctive group of progressive optic neuropathies, afflicting over 60 million people and resulting in 8.4 million cases of irreversible blindness globally^1^. To date, although multiple mechanisms have been proposed to describe the processes leading to glaucoma^2^, it is agreed that vision loss in glaucoma results principally from the loss of RGCs via apoptotic cell death^3^.

With the emergence of techniques such as DARC (Detection of Apoptotic Retinal Cells)^4^ and Adaptive Optics confocal Scanning Laser Ophthalmoscopy (AO-cSLO)^5^, permitting the in vivo visualisation of retinal cell populations approaching single cell-resolution, there is renewed interest in identifying disease specific patterns in retinal cell loss to permit early diagnosis of diseases such as glaucoma. Initial attempts to determine RGC subpopulations at greatest susceptibility to glaucomatous injury have been focusing on cell morphology, and have stratified RGC populations in retinal histological samples based on cell size. Early work in this field suggested that larger RGCs were more susceptible to early loss than smaller RGCs in monkeys with monocular experimental glaucoma^6–8^ and in patients with elevated intraocular pressure (IOP)^9^. Based on these findings, the authors postulated that larger RGC subpopulations, such as those of the magnocellular pathway, would be lost early in the disease process.

A growing body of evidence, however, suggests that the visual response to motion is not solely dependent on the magnocellular pathway^10^, and several limitations of this work were subsequently recognised by Morgan et al.^11^, including the hypothesis that soma and axon size may be dynamic and change in response to injury; the inhomogeneous distribution of RGCs of different sizes in the retina, limiting the validity of conclusions drawn from manually sampled retinal sectors; and the labelling technique used (Nissl staining), which requires subjective assessments to distinguish between amacrine and RGC labelling. Indeed, adding to this controversy, subsequent work reported that larger RGCs were found to be no more susceptible to loss than smaller RGCs, after assessment of an RGC population (n = 1282 cells) in an ocular hypertension model in monkeys. In addition, RGC shrinkage may contribute to previous contrary observations, with larger RGCs expected to change size to a greater extent than smaller RGC^12,13^.

Recently there are several reports that the large melanopsin-expressing RGCs are more resistive to degeneration in rodent models of optic nerve injury^14^ and chronic ocular hypertension^15^, than smaller RGCs. In a final complication, there are also reports of RGC size increase in response to injury in rodent models of optic nerve injury^16,17^ and glaucoma^18^, based on retinal sampling approaches (n ~ 120 000 RGCs). This increase in cell size has been attributed to increased gene expression in response to injury or an attempt to increase dendritic coverage and compensate for recently lost RGC neighbours^19–21^.

In order to address this controversy, we sought to apply recent advances in maximising information extraction from RGC populations in retinal whole mounts^22,23^ to evaluate the changes in RGC size distribution in three well-established rodent models of optic nerve injury: the Morrison’s ocular hypertension (OHT)^24^, murine optic nerve crush (ONC) and partial optic nerve transection (pONT) models^25,26^. OHT and pONT models were induced in rat, while ONC was induced in mice. In contrast to previous studies based on sampling approaches, we evaluated the change in size of the whole Brn3a-positive RGC population at multiple time points over the natural history of these models. To achieve this, the morphology of over 4 million RGCs was assessed from a total of 74 retinae to glean novel insights from these datasets^22^. Finally, to evaluate whether sampling a small area of the RGC population from retinal wholemounts (mimicking a typical AO-cSLO field of view) can provide sufficient information (spatial and morphological) to distinguish between glaucoma and naive retina, a bootstrapping approach was employed. Bootstrapping is a powerful statistical technique involving random sampling with replacement in order to assign measures of accuracy (confidence intervals prediction error etc.) to sample estimates^27^. Here, this technique was used to predict the diagnostic power of a randomly selected 0.5 mm^2^ subset of the RGC population in each disease model.

## Methods

### Animals

All animal experiments were performed with procedures approved by the U.K. Home Office and the KU Leuven institutional ethical committee, and are in accordance with the ARVO Statement for the Use of Animals in Ophthalmic and Vision Research. A pre-existing data set consisting of, in total, 67 adult male DA rats (weighing 150 to 200 g) (Harlan Laboratories, Indianapolis, IN, USA) and 7 adult male C57BL/6 J mice (15 to 25 g) (Charles Rivers Laboratories, Wilmington, MA, USA) was used in this study^22,28^. Animals were housed in an air-conditioned, 21 °C environment with a 12 h light–dark cycle (140–260 lx), where food and water were available ad libitum.

### AAV-GFP vector administration

Two DA rats and three C57BL/6 J mice received a unilateral intravitreal administration of 2 or 5 µL of adeno-associated virus (AAV)-based mutAAV2/2(Y444F)-CMV-eGFP vector (titre: 6 × 10^11^ GC/ml) (Leuven Viral Vector Core). Procedures were conducted under general anaesthesia using a mixture of 75 mg/kg ketamine (Anesketin, Eurovet, Clichy, France) and 0.5 mg/kg medetomidine (Pfizer Animal Heath, New York, NY, USA) for rats, and 75 mg/kg ketamine and 1 mg/kg medetomidine for mice. Animals were sacrificed 14 days post injection of the AAV vector, and retinas were flat-mounted prior to Brn3a and GFP double immunohistochemistry.

### Ocular hypertension (OHT) model

As described in the original paper^22^, ocular hypertension was surgically induced in the left eye of 23 DA rats. Procedures were conducted under general anaesthesia (as described above). Briefly, 50 µL of hypertonic saline solution (1.8 M) was injected into two episcleral veins, using a syringe pump (50 µL/min; UMP2; World Precision Instruments, Sarasota, FL, USA). A propylene ring with a 1 mm gap cut from the circumference was placed around the equator to prevent injected saline outflow from other aqueous veins. The IOP from both eyes of each rat was measured before the procedure and after at regular intervals, using a TonoLab tonometer (Tiolat Oy, Helsinki, Finland) under inhalational anaesthesia (0.4% isoflurane in oxygen). Animals were sacrificed 7, 21, 56 and 84 days after unilateral IOP elevation and retinas flat-mounted prior to Brn3a immunohistochemistry.

### Partial optic nerve transection (pONT) model

As described in the original paper^28^, partial optic nerve transection was conducted in the left eye of 26 DA rats. Under general anaesthesia, an incision was made in the superior conjunctiva, and the ON sheath was exposed. A longitudinal slit was next formed in the dura mater to expose the ON and a 0.2-mm cut was made in the dorsal ON, 2 mm behind the eye using an ophthalmic scalpel with steel cutting guard. Damage to major ophthalmic blood vessels was avoided and verified at the completion of surgery by ophthalmoscopy. Animals were sacrificed 3, 7, 21, and 56 days after pONT induction and retinas flat-mounted prior to Brn3a immunohistochemistry.

### Optic nerve crush (ONC) model

Intraorbital optic nerve crush was performed in the left eye of two C57BL/6 J mice using a previously described technique^26^. Briefly, a temporal incision was made in the conjunctiva and the exposed optic nerve was crushed 1 mm from the globe using cross-action forceps (no 11262–30, Fine Science Tools, Heidelberg, Germany) for 5 s. Funduscopic examination was performed before and after this procedure to assess integrity of retinal perfusion. Animals were sacrificed 4 and 7 days post ONC respectively. A separate cohort of two untreated animals served as controls.

### Immunohistochemistry and confocal microscopy

Immunostainings for Brn3a, GFP and RBPMS on retinal whole-mounts were completed as described previously^22,28,29^. The primary antibodies used are: mouse anti-Brn3a for single labelling (1:500, Merck Millipore, Darmstadt, Germany); goat anti-Brn3a for co-labelling with GFP or RBPMS (1:750, Santa Cruz, Dallas, TX, USA); rabbit anti-GFP (1:10,000, in-house made); and rabbit anti-RBPMS (1:200, PhosphoSolution, Aurora, CO, USA). Immunolabeled RGCs were examined under confocal microscopy (LSM 710, Carl Zeiss MicroImaging GmbH, Jena, Germany). Each retinal whole mount was imaged as a tiled z-stack at × 10 magnification, which was used to generate a single plane maximum projection of the RGC layer in each retina for subsequent analysis. Retinal image acquisition settings were kept constant for all retinas imaged, allowing comparison of Brn3a expression in each experimental group as previously described^30^. Each whole mount image was manually orientated, using in vivo cSLO imaging of retinal vasculature as a reference, so that the superior retina was towards the top of the image.

### Calculating the rate of RGC loss

To determine the half-life of RGC loss, RGC densities gated by nuclear size over the natural history of the pONT and OHT models were fit to a one-phase exponential decay model with plateau (p) as previously described (Eq. 1)^22^.

1 $$Y = (Y_{0} - P)e^{{ - \left( {kx} \right) + P}}$$

### Bootstrapping of retinal wholemounts

To estimate whether differences between disease (OHT and pONT models) and healthy (controls) populations can be identified by observing only a small subset of the flat-mounted retina, a bootstrapping approach was used. This process consisted of the following steps:

1. Retinas from control and disease groups was randomly drawn from the population with replacement.
2. A rectangular window of fixed size (0.5 mm^2^) was randomly placed on each retinal wholemount. The position of this window was drawn from a uniform distribution with limits equal to the image size minus the size of the rectangular window. Successive draws could therefore produce successive rectangular windows that overlap, however, all rectangular windows were of the same size.
3. As retinal wholemounts had irregular shapes, to avoid selecting empty sets (which are not of physiological relevance) rectangular windows were required to contain a minimum of 100 RGCs. This value was chosen in order to eliminate regions containing only background (no retina), but not bias against regions of severe disease which contained fewer cells. Retinal area contained within each rectangular window was determined by calculating the fraction of the rectangular window contained within a previously constructed binary mask of the wholemount retinal area.
4. Once selected, statistics of interest (including, nearest neighbour distance, regularity index, mean RGC area and mean absolute deviation) were calculated and results recorded for each window of observation.
5. This process was repeated 100,000 times for each of the three groups of retina to provide a reasonable approximation of the exact case control. Differences between disease and experimental conditions were then calculated in each case before violin plots were constructed using ggplot2^31^ and ROC curves using pROC^32^ R packages, respectively.

### Morphological segmentation of RGCs from retinal wholemounts

Automated quantification of Brn3a-labelled RGCs in retinal whole-mounts was completed as described previously^22^. Morphological segmentation of RBPMS^+/^Brn3a^+^ double-labeled RGCs was completed using the MorphlibJ Morphological segmentation plug-in for ImageJ FIJI^31^. Briefly, this technique requires three components: an RBPMS labelled image to segment, a binary mask of the extent of RBPMS labelling in the retina, and a series of markers denoting the positions of each cell (derived from the Brn3a channel using aforementioned Brn3a algorithm). The RBPMS mask was obtained by enhancing local area contrast (49 pixel radii), followed by a one pixel radii median filter to remove small pixel noise, an Otsu intensity threshold to create an image mask, and finally a watershed algorithm to separate touching objects. The RBPMS-labelled image was subject to the same local intensity enhancement and median filter, prior to running the morphological segmentation script. Once complete, the positon (x,y) and area of Brn3a^+^ and RBPMS^+^ RGCs were recorded separately. Using the spatstat package in R^32^, Brn3a and RPBMS channels were used to establish point pattern processes marked with cell areas in each case. Then, cross nearest neighbour distances between Brn3a and RBPMS were calculated to determine which Brn3a-labelled measure corresponded to which RBPMS^+^ RGC. To simplify the analysis, RGCs with multiple nearest neighbours (typically < 5% of total population) were excluded from further analysis. Matched RBPMS^+^ and Brn3a^+^ areas were plotted and correlations determined using Spearman’s correlation coefficient. Linear regression was used to determine the CN ratio for each retina.

### Statistical Analysis

All data were analysed using GraphPad Prism 8.4.3 (GraphPad Software, Inc., La Jolla, CA, USA; www.graphpad.com) as appropriate. Data were presented as means ± SE and *p* < 0.05 was considered significant. 95% Confidence intervals for Spearman’s rho were calculated according to methods described previously^33^.

## Results and discussion

### Can nucleus size be used as a surrogate of cell size?

RGCs were immunohistochemically labelled with Brn3a in retinal whole mounts^23,34^, and RGC populations were segmented using a previously validated algorithm that was modified to additionally record the nuclear area of each RGC^22^. Cell soma size is believed to correlate with nucleus size in a number of cell types^35^, including RGC populations, as described in the ONC and DBA/2 J glaucomatous mouse models^18,36^. To confirm that the RGC cytoplasmic-to-nuclear (CN) ratio held true for RGCs in our data set, two Dark-Agouti (DA) rats and three C57Bl6/J mice received a unilateral intravitreal injection of a AAV2/2-GFP vector, which results in cytoplasmic expression of GFP in transduced cells. After co-labelling with Brn3a, eight 500 µm × 500 µm regions from each retina were selected at fixed eccentricities from the optic nerve head (ONH). A manual observer identified cells that were co-labelled (GFP^+^ Brn3a^+^) (Suppl. Fig. 1), such that 300 total cells were included in mouse and 368 in rat retinae. Comparison of nuclear to cytoplasmic area revealed a significant positive correlation between nucleus and cytoplasmic area in both the murine retina (Spearman’s R = 0.6858, CI_95%_ range = 0.03) and rat retina (Spearman’s R = 0.6281, CI_95%_ range = 0.03), giving rise to cytoplasmic-to-nuclear ratio of 2.00 ± 0.03 (murine) and 2.43 ± 0.03 (rat), respectively. Together, these data suggest that Brn3a-determined RGC nuclear size can be used to inform about RGC soma area in the naive rodent retina.

### Is cytoplasmic to nuclear ratio maintained in response to injury?

**Figure 1:**
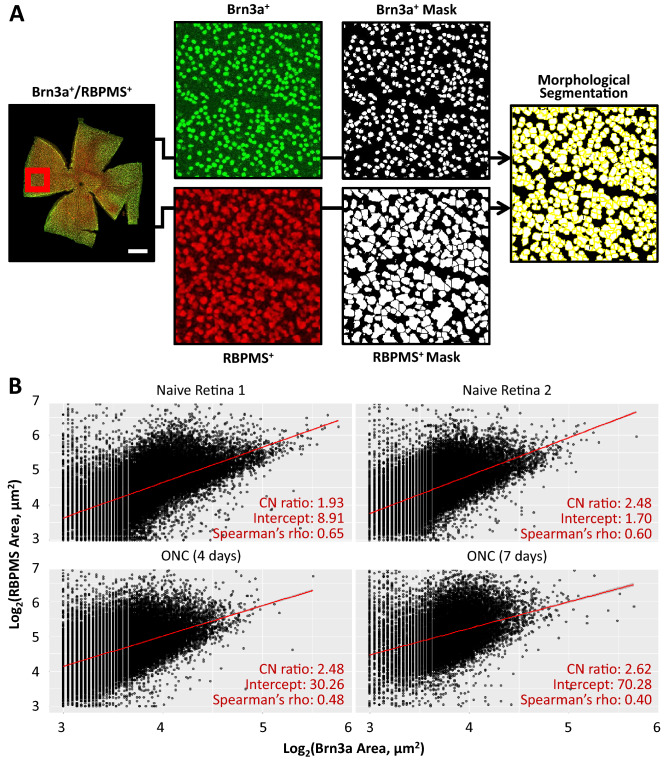
RBPMS and Brn3a colabelling of murine retinal ganglion cells in naive retina and after induction of optic nerve injury, using an established optic nerve crush model. (**A**) Illustration of the protocol for automatically measuring RGC cytoplasmic (RBPMS) and nuclear (Brn3a) area as described in the text. (**B**) Plots of Brn3a labelled area versus RBPMS labelled area in each retina with linear regression fit shown in red. Matching between Brn3a and RBPMS areas was achieved by calculating the cross-nearest neighbour distance between populations as described in the text.

A limitation of using RGC nuclear size as a surrogate of cell size is the extent to which the CN ratio is maintained during injury induction. To address this possibility, a total of 130.072 RGCs, from naive mice (82.746 cells) and animals subjected to ONC (4d and 7d post-injury, 31.455 and 15.871 cells, respectively) were analysed. Wholemout retinae were co-labelled with Brn3a and RBPMS, *i.e.* one nuclear and one cytoplasmic marker specific for RGCs^37^. A morphological segmentation algorithm was used whereby the location of Brn3a^+^ RGCs was first isolated using previously established methods, before these points were used as markers for subsequent isolation of the RBPMS derived cytoplasmic area (Fig. 1A)^31^. A limitation of this approach is that no information will be obtained for RBPMS^+^/Brn3a^−^ RGCs (~ 3% of total RGC population), which include the mRGC population^38–41^.

The RBPMS^+^/Brn3a^+^ RGC areas under each injury condition were plotted and correlation coefficients determined in each case to establish the extent of CN ratio preservation on injury induction (Fig. 1B). In naive retina, RBPMS/Brn3a derived CN ratios showed excellent agreement with the AAV-GFP method (Spearman’s correlation coefficients of r = 0.60 and 0.65 respectively, CI_95%_ ranges: 0.001 & 0.008). After ONC injury, correlation between cytoplasmic and nuclear area remained similar to naive retina at day 4 (Spearman’s correlation coefficient = 0.58, CI_95%_ range = 0.004), however reduced by day 7 post-ONC (Spearman’s correlation coefficient = 0.40, CI_95%_ range = 0.01), suggesting that injury has the potential to moderately influence the CN ratio, although a substantial correlation between cytoplasmic and nuclear area remained. The CN ratio of RGCs after ONC was then determined using linear regression and found to be in agreement with previous AAV-GFP data (data not shown). The Spearman’s rho suggests that approximately 50% of the variability in RGC nucleus size can be explained by RGC cytoplasmic area, suggesting nuclear area is a moderately strong predictor of cell size. The remaining variability can be explained by differences in CN ratio between RGC subtypes and the potential for cell shrinkage in response to injury associated stress, interesting topics for future investigation.

### RGC size changes dynamically in response to injury

**Figure 2:**
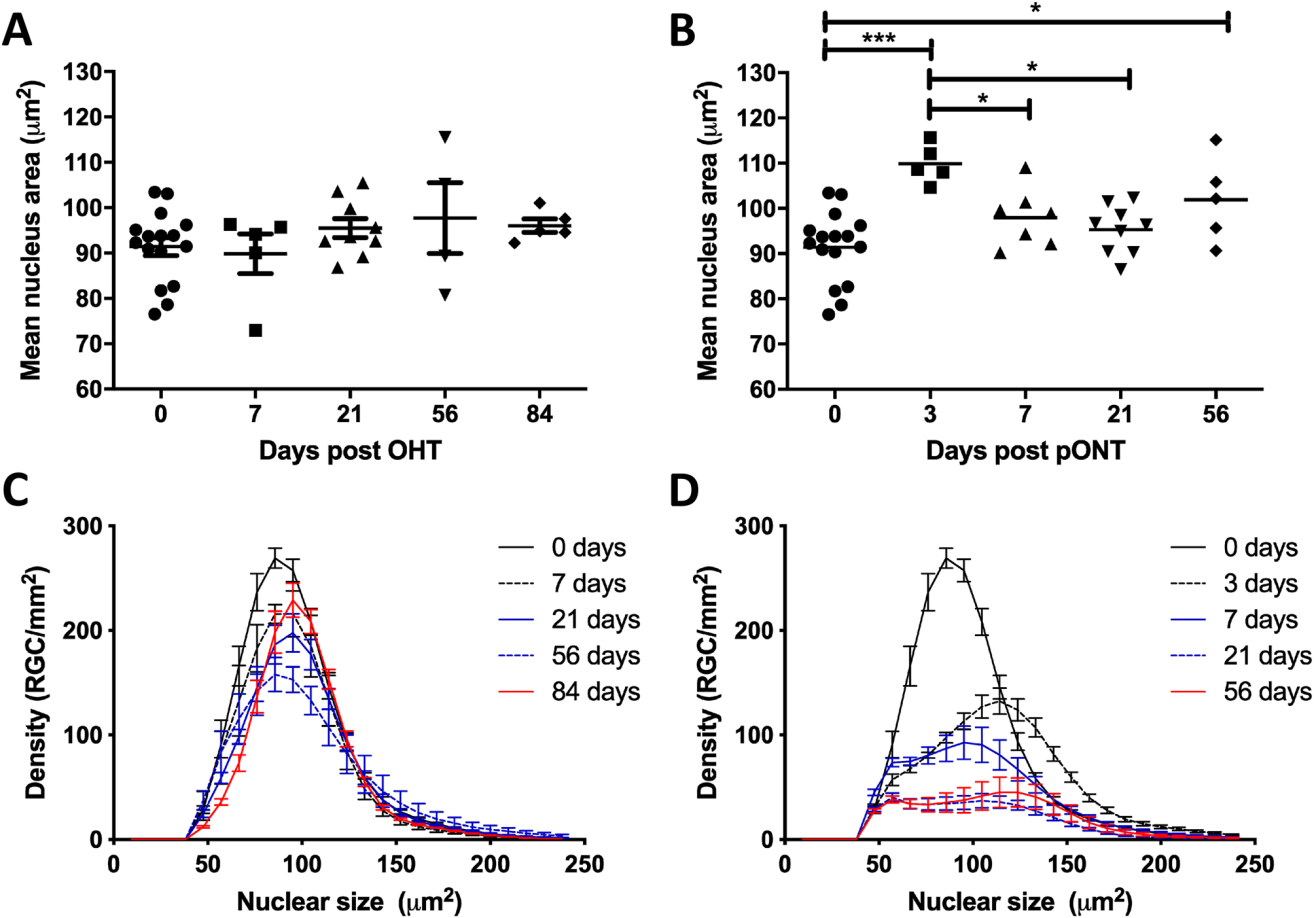
The distribution of RGC nucleus area changes over the course of the OHT (**A**, **C**) and pONT (**B**, **D**) models. (**A**) Average RGC nucleus area does not significantly change over the course of the OHT model. (**B**) However, a significant increase in nuclear area is observed 3 days after pONT induction, followed by a reduction in area from 7 days post pONT (One-way ANOVA, Tukey post-hoc test, *p < 0.05, ***p < 0.001). Data points represent individual animals, to illustrate inter-animal variability. (**C**, **D**) Overall, the RGC density declines and the distribution of RGC density by nucleus area shifts to the right over the course of the (**C**) OHT model and (**D**) pONT models, although this trend is more subtle in the OHT model.

Having established that mean RGC nucleus area is an objective parameter, we next used the algorithm to assess an established dataset of Brn3a-labelled DA rat whole retinal mounts, comprising images over the natural history of the OHT (0, 7, 21, 56 and 84 days) and pONT models (0, 3, 7, 21, 56 days) (Fig. 2A,B)^22^. While no significant change in mean RGC nucleus area was observed over the natural history of the OHT model (Fig. 2A), a significant increase in mean RGC nucleus area was observed at 3 days following pONT induction (Fig. 2B), followed by a reduction in area towards baseline levels from 7 days post pONT onwards (one-way ANOVA with Tukey post hoc tests, *p* < 0.05).

To further elucidate these trends, mean histograms of RGC density binned by nuclear area (25 bins was chosen arbitrarily, each 9.52 µm^2^ in size) were plotted at each time point following OHT (Fig. 2C) or pONT (Fig. 2D) induction. A significant reduction in the density of RGC nuclei between 57 and 105 µm^2^ (unmatched two-way ANOVA with Bonferroni post hoc test *versus* naive controls, *p* < 0.05) was recorded, starting at 7 days post OHT model induction and persisting until day 84 (Suppl. Table 1). A similar reduction in the density of smaller RGCs was also observed over the natural history of the pONT model (57 to 133 µm^2^, Suppl. Table 1), with no significant change in the density of the largest (> 133 µm^2^) RGC populations. Additionally, after three days of pONT model induction, a transient but significant increase in the density of larger RGCs was observed, of size between 133 and 143 µm^2^ (Suppl. Table 1). A possible explanation for the perceived increase in the density of larger RGCs is that these cells have responded to injury by increasing in size, supporting previous suggestions that RGC size is dynamic in response to injury^20,21^ and that, to an extent, increases in cell size reflect changes in metabolic function, such as increasing protein content, mitochondrial mass and mitochondrial membrane potential when energy demands on cells are increased^41,42^.

### Some RGC types are more susceptible in rodent models of retinal injury

**Figure 3:**
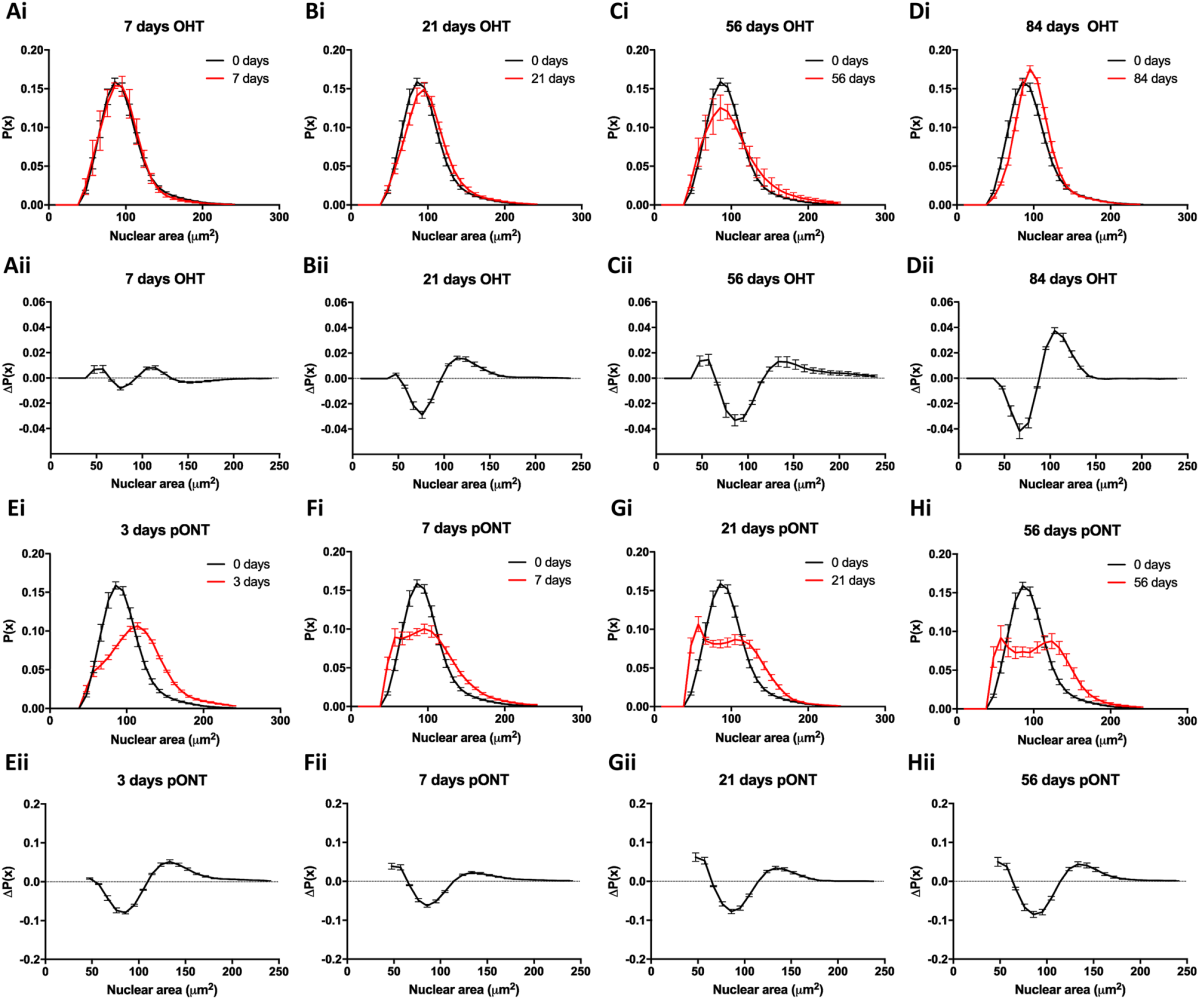
(**A**–**D**) Assessment of RGC nucleus size probability distribution functions (i), and difference *versus* control condition (ii), over the natural history of the OHT model. (**E**–**H**) Assessment of RGC nucleus size probability distribution functions in the pONT model (i), and difference *versus* control condition (ii), over the natural history of the pONT model. Mean ± SE.

To further investigate size dependent biases in the reduction in RGC density over the course of the OHT and pONT models, probability distribution functions (PDF) were constructed to monitor the change in the probability of observing RGCs with each nuclear area over the course of each model (Fig. 3; Suppl. Fig. 2). In the OHT model, the reduction in RGC density is accompanied by a general shift in the RGC PDF to larger nuclear sizes by 84 days (Fig. 3D), when intraocular pressure (IOP) and RGC apoptosis have largely returned to basal levels^22^. This observation suggests that either RGCs with smaller nuclei are preferentially lost in this model, or that a subset of the RGC population increases in size in response to this injury. Over the course of the milder OHT model, a transient subtle increase in the probability of observing smaller (< 60 µm^2^ nuclear area) is also observed. One interpretation of this observation is that a subpopulation of RGCs become smaller in response to injury. This observation is supported by previous findings that RGC shrinkage occurs as a precursor to apoptosis induction^36^. These patterns were subtle owing to the relatively mild nature of the OHT model and when the same analysis was applied to the pONT model (Fig. 3E–H), more pronounced shifts in the PDF were observed.

Indeed, 56 days after pONT induction, a significant reduction in the probability of observing smaller RGCs was detected, in a similar fashion as reported for the OHT model. Interestingly, the PDF of the pONT model shifts markedly to the right after three days of model induction, indicative of a preferential loss of smaller RGCs and/or a proportion of the RGC population becoming larger in size in response to injury (Fig. 3E). By 7 days after model induction, the PDF develops a clear biphasic character, with an increase in the probability of both smaller and larger RGCs being observed. This character was similar to the pattern of RGC loss observed in the OHT model until 56 days (Fig. 3C). As cell loss in the pONT model is continuing at 56 days, the biphasic character has not yet resolved into the same pattern of cell loss as observed in the OHT model after 84 days.

One interpretation of these data is that in response to injury (pONT), RGCs dynamically react with a subset of the population becoming smaller in response to injury and a second population increasing in size, as evidenced by an increase in the density of smaller and larger RGCs after model induction *versus* baseline (Fig. 2C,D). Together, these results suggest a dynamism in RGC size in response to injury with smaller RGCs (< 100 µm^2^ nuclear area) being more susceptible to loss than larger RGCs. RGC shrinkage prior to loss may be indicative of these cells undergoing apoptosis^36^. Of note, besides dynamic changes in cell size, selective loss of RGCs likely also contributes to these observations.

### Is RGC loss susceptibility dependent on size and/or position?

So far, we have presented evidence to suggest that subsets of the RGC population dynamically respond to optic nerve injury, and that similarities in the size dependence in RGC susceptibility to loss exist between OHT and pONT models. As smaller RGCs have been suggested to preferentially localise to the centre of the rodent retina^17^, and we and others previously described heterogeneous RGC loss across the retina in OHT and pONT models (^22^, reviewed in^43^), we sought to determine whether the selective loss of smaller RGCs observed was a result of the inhomogeneous distribution of small RGCs in the rodent retina. By correlating RGC nuclear area with distance from the optic nerve head (Suppl. Fig. 3), a significant positive correlation was observed in naive retina (Spearman’s correlation coefficient = 0.26, CI_95%_ range = 0.008). This was lost at 56 days after pONT (Spearman’s correlation coefficient = 0.04, CI_95%_ range = 0.001) and substantially weakened at 84 days after OHT model induction (Spearman’s correlation coefficient = 0.18, CI_95%_ range = 0.006). These data suggest that in the healthy retina, smaller RGCs are more likely to be localised towards the ONH than larger RGCs, which could be a result of steric hindrance close to the ONH due to increasing RGC packing density, greater energetic demands placed on RGCs with longer axons further from the ONH, or differences in RGC dendritic field size between central and peripheral retina. At 56 days after pONT and 84 days of OHT, however, this preferential location in the central retina seems to be lost.

To evaluate whether the distance from the ONH influenced the susceptibility of RGC loss by size, the differences in PDF of RGC populations at 84 days after OHT and 56 days after pONT were evaluated and compared to naive controls (Suppl. Fig. 4). The distribution of RGC loss was observed to be similar for all eccentricities from the ONH (where A-O represent concentric 0.3 mm diameter non-overlapping rings centred on the ONH, illustrated in Suppl. Fig. 4P) in both OHT (i) and pONT (ii) models, suggesting that the pattern of RGC loss was maintained at each retinal eccentricity. Thus, although susceptibility to cell death seems to be related to cell size, localization of the RGC in the retina does not appear to underlie this differential susceptibility.

### Smaller RGCs are more prone to primary degenerative loss than larger RGCs

**Figure 4:**
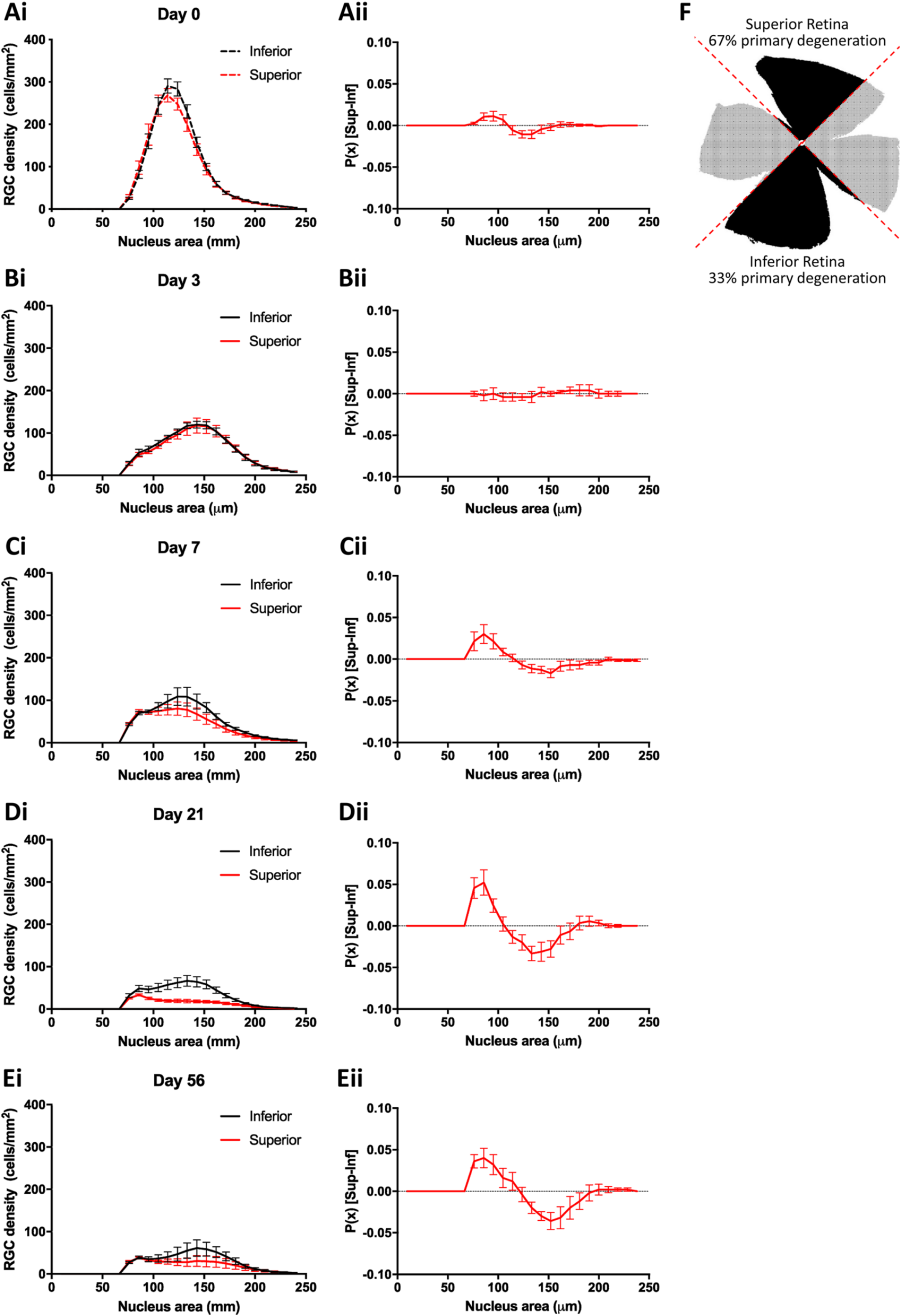
The size dependence of RGC loss in the pONT model is related to susceptibility to primary and secondary neurodegeneration. (**Ai**–**Ei**) PDF of RGC soma area from paired superior vs inferior retinal quadrants at each timepoint. (**Aii**–**Eii**) Difference between superior and interior PDFs from (**Ai**–**Ei**). At first, retinal neurodegeneration is comparable in both superior and inferior retinal quadrant regions. As the model progresses, however, a greater proportion of smaller RGCs are observed in the superior than inferior retina, suggestive that RGCs becoming smaller precedes primary degeneration or that larger RGCs are more susceptible to secondary degeneration. No such trend was observed in the OHT model (data not shown), most likely due to the milder, more chronic nature of this model. (**F**) Schematic representation of retinal regions more prone to primary vs secondary neurodegeneration in the pONT model.

Previously, we reported that pONT leads to a partial spatial separation of primary and secondary neurodegenerative processes, with approximately ~ 67% of RGC loss in the super retinal quadrant occurring through primary degenerative processes, compared to only ~ 33% in the inferior retinal quadrant^22^. To investigate whether RGC nuclear size correlated with primary or secondary degeneration processes, retinas subjected to pONT were spatially segmented into superior and inferior quadrants (Fig. 4; Suppl. Fig. 5), from which superior and inferior PDFs were constructed at each time point. As the proportion of primary versus secondary degeneration is different in the superior and inferior retina (with more primary neurodegeneration (due to directly severed axons) in the superior retina and more secondary neurodegeneration in the inferior retina) in the pONT model (Fig. 4F)^22,44^, a difference in PDF between superior and inferior retina may indicate a bias in RGC size and differential susceptibility to primary or secondary degeneration. At baseline (Fig. 4B) and 3 days after pONT induction (Fig. 4C) there was no apparent superior/inferior selectivity in the loss of RGCs. A possible explanation for this observation is that during this time, RGCs are lost only by primary degenerative processes that occur shortly after injury. After this time, a noticeable shift in PDF was observed from 7 days post pONT induction, with a greater probability of smaller RGCs being present in the superior than inferior retina. One explanation for this observation is dynamism in the RGC population, either as a result of RGCs shrinking in the superior retina or expanding in the inferior retina in response to injury. Alternatively, this data could be interpreted to suggest that larger RGCs are more susceptible to primary degenerative processes.

**Figure 5:**
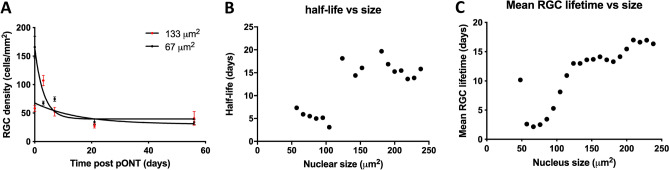
Relationship between nucleus area and rate of loss (half-life) over the course of model. RGC densities binned by nucleus area were plotted over the natural history of the OHT and pONT models before fitting to exponential decay equation (**A**). In both (**B**) OHT and (**C**) pONT models, smaller RGCs typically have shorter half-lives than larger cells, suggesting preferential loss of smaller RGCs from the retina.

To resolve these possibilities, RGC density, binned by nuclear size was fit to a single-phase exponential decay model (Eq. 1) to establish the half-life of each RGC subpopulation over the history of OHT and pONT models (Fig. 5). In both OHT (Fig. 5A) and pONT (Fig. 5B) models, smaller RGCs were found to be lost more rapidly than larger RGCs, suggesting that Fig. 4 illustrates a subset of RGCs in the superior retina reducing in size prior to rapid cell loss.

### Assessing the feasibility of using disease-associated changes in RGC spatial distribution and size as novel diagnostic indicators

**Figure 6:**
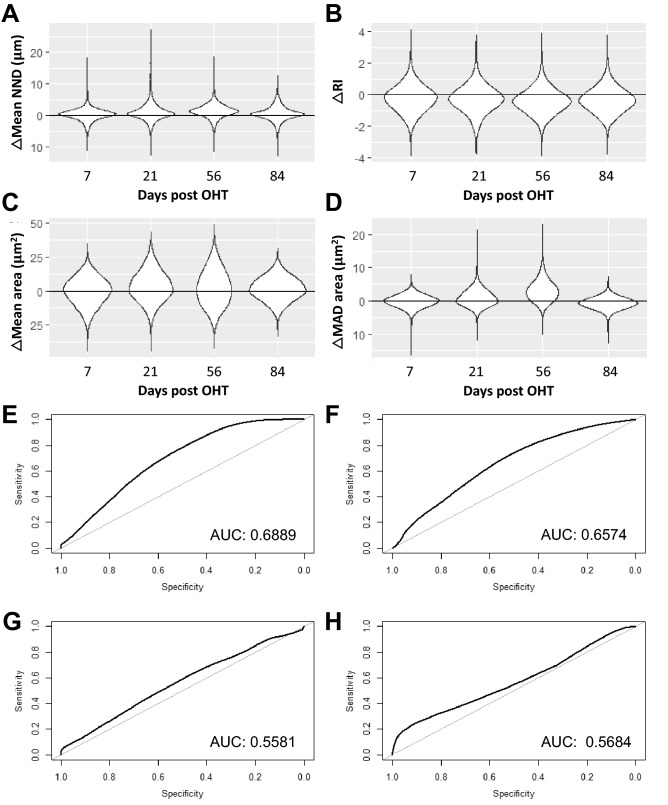
Determination of the diagnostic utility of subsampling the RGC population in the OHT model using a Spatial Bootstrapping versus naive controls. (**A**–**D**) Difference between OHT and Naive retina randomly subsampled 100 000 times as described in the text and corresponding ROC curves (**E**–**H**). Results compared include (**A**, **E**) Nearest Neighbour Distance (NND), (**B**, **F**) Regularity Index (RI), (**C**, **G**) Mean RGC size and Mean absolute deviation in RGC size (**D**, **H**). A table with the median and 95% confidence intervals for the bootstrapping results has been added in supplement (Suppl. Table 2).

**Figure 7:**
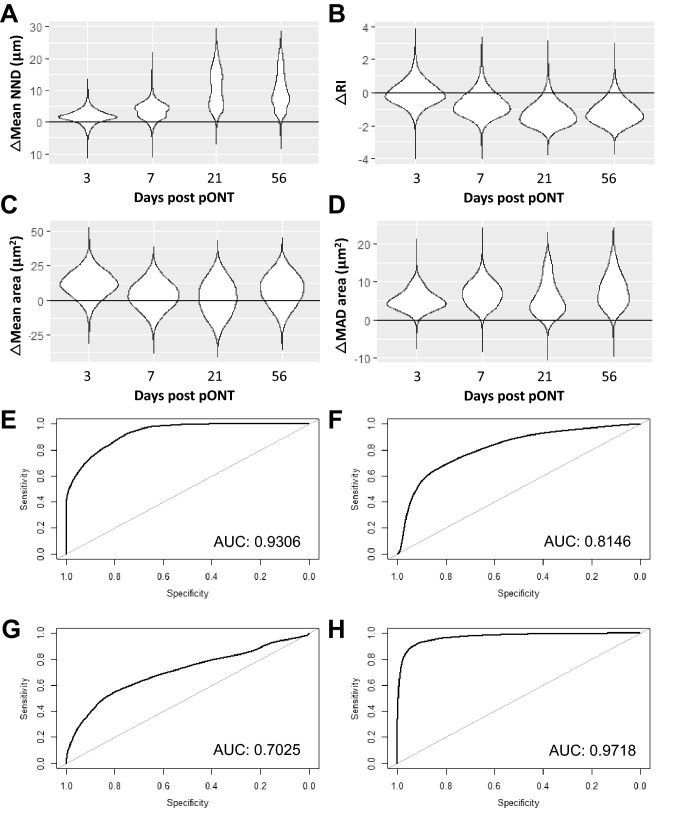
Determination of the diagnostic utility of subsampling the RGC population in the pONT model using a Spatial Bootstrapping versus naive controls. (**A**–**D**) Difference between pONT and Naive retina randomly subsampled 100 000 times as described in the text and corresponding ROC curves (**E**–**H**). Results compared include (**A**, **E**) Nearest Neighbour Distance (NND), (**B**, **F**) Regularity Index (RI), (**C**, **G**) Mean RGC size and Mean absolute deviation in RGC size (**D**, **H**). A table with the median and 95% confidence intervals for the bootstrapping results has been added in supplement (Suppl. Table 2).

Adaptive Optic confocal Scanning Laser Ophthalmoscopy (AO-cSLO) and Detection of Apoptotic Retinal Cells (DARC) represent emerging techniques for the non-invasive visualisation of RGCs in the human retina at the single cell resolution. A limitation of these approaches is the field of view, which presently only permits the visualisation of a few hundred RGCs per image in the case of AO-cSLO. To evaluate whether sampling of the RGC population in this manner can provide sufficient information (spatial and morphological) to distinguish between glaucoma and naive retina, a bootstrapping approach was employed, whereby retinae were divided into three groups (naive, OHT and pONT) and randomly sampled 100 000 times, in each case using a 0.5 mm^2^ rectangular window of observation. A total of four measures were recorded from each observation window: average nearest neighbour distance (NND), regularity index (RI), mean RGC area and mean absolute deviation (MAD) of RGC area. Here, NND is defined as the average distance between each RGC and its nearest neighbour in the retinal mosaic. RI comprises a spatial statistic derived from the frequency distribution of NNDs, and is calculated by dividing the mean NND by the standard deviation of the NND of a population within a sample field. The MAD of RGC area is measure of the variability in a dataset, and is defined as the average distance between each data point and the mean RGC area. Results from OHT and pONT bootstraps were compared to results from naive retina by the construction of ROC curves, and the AUCs were compared. Furthermore, mean differences in OHT and pONT parameters *versus* naive measures were compared (Figs. 6, 7, respectively).

The OHT model, which was the more-mild of the two models investigated, recorded AUCs of 0.69, 0.66, 0.56 and 0.57 for NND, RI, mean cell size and MAD cell size, respectively (Fig. 6). The pONT model by contrast recorded AUCs of 0.93, 0.81, 0.70 and 0.97 for NND, RI, mean cell size and MAD cell size, respectively (Fig. 7). These data suggest that each of these measures may have diagnostic utility in identification of more severe retinal injury. Particularly intriguing is that the latter three measures are independent of RGC density, which is important as variation in the number of RGCs between healthy individuals is well documented^45,46^. Future studies may be able to increase the diagnostic power of this technique by spatially restricting the position of the observation window to a particular region of the retina (i.e. 1 – 1.5 mm from the ONH) or by considering more complex spatial patterns of RGC populations, which may further increase the diagnostic power of this technique. This has important implications for retinal imaging technologies, where wide-field, whole retinal visualization is currently difficult without pupillary dilation and time-consuming approaches. In summary, these data suggest spatial measures of RGC population subsets may have diagnostic utility in conditions causing retinal injury, with utility increasing with injury severity.

## Conclusion

In summary, this study highlights the importance of understanding patterns of cell loss in retinal neurodegeneration. Although the models used in the study are based on glaucoma and optic nerve disease, as secondary neurodegenerative processes are increasingly thought to play an important role in other disorders of the CNS, these findings may have wider implications for other neurodegenerative conditions.

This work suggests that dynamic changes in nucleus size – here used a as a proxy for cell size – do relate to cell fate after glaucomatous injury. Or alternatively, RGCs of different size die differentially at different time points post injury. This study indicates that RGC size is dynamic in response to injury, with smaller RGCs being found to be more susceptible to primary degeneration, compared to larger RGCs which in turn are more prone to secondary degenerative processes. Secondly, extremes of size in the RGC population appear to be most resistant to loss in both pONT and OHT models, which could be attributed either to a greater innate resistance of these RGC subpopulations to loss in these models, or to the dynamic nature of RGC cell size in response to injury. In support of the second hypothesis, and thirdly, although some RGC shrinkage occurs in response to injury and could be indicative of early apoptosis, the majority of RGCs tend to increase in size on injury, which may be associated with upregulation of cell processes with the goal of resisting cell loss^41^.

Using this information, the following model of RGC loss in response to optic nerve injury is proposed, whereby;

1. In response to injury, RGCs dynamically increase in size. This could reflect an attempt of these cells to increase protein and mitochondrial content in order to survive an environment with greater (or altered) energy demands. Inhibition of oxidative phosphorylation, induction of oxidative stress and mild uncoupling of mitochondrial membrane potential may converge into a protective cellular response that leads to cell expansion. The latter then reflects higher mitochondrial mass or increased mitochondrial membrane potential, leading to higher rates of transcription and translation and ATP generation^41,42^. This process may also include an increase in pro-survival gene expression^47^, activation of additional pro-survival pathways such as autophagy, reported to increase RGC survival in response to injury^48^. Autophagy upregulation has recently been suggested to result in increased cell size among jurkat T-lymphocytes^49^.
2. RGCs unable to meet the increased energy demands of the injured environment, begin to undergo apoptosis, a process characterised by cell shrinkage prior to loss. Combined changes in ion channel fluxes, plasma membrane transporter activity, and water flux – all highly energy demanding processes that may be compromised under pathological circumstances –, as well as cytoskeletal reorganization, are believed to underlie this cell shrinkage. By setting intracellular ionic strength conditions, this so-called apoptotic volume decrease regulates caspase and nuclease activity and thereby critically modulates the decision of a cell to undergo apoptosis^50–53^. RGC may also decrease their cell size due to a loss of growth factor support from their target neurons^54^, which will eventually also lead to their death.
3. As RGCs shrink prior to apoptotic loss, this may be responsible for the apparent resistance of the very smallest RGCs to degeneration (< 56 µm^2^), as continued loss of RGCs through this process acts to replenish this population of cells despite their continual loss.
4. Over time, larger RGCs – which initially increased in size as a result of the stress response induced by the injury – begin to either return to their original size as the injury abates, or begin to succumb to secondary degenerative processes, perhaps as a result of increased mitochondrial, oxidative and excitotoxic stress.

Previously, the increased survival of melanopsin-expressing RGCs (mRGCs) – which are a subtype of large RGCs – has been suggested to be a result of an as yet unidentified property of these cells that preserves them from injury^15^. Notably, Brn3a does not label mRGCs^38–41^, suggesting that the properties of larger RGCs that enable them to resist degeneration are common to other larger RGC subtypes also, and the result of melanopsin-independent effects such as a greater number of mitochondria^55^. Future studies will have to disentangle the cellular and/or molecular basis for this differential resilience of RGC subtypes, e.g. by studying the transcriptional, physiological, and morphological characteristics of the at least 46 subtypes of RGCs identified thus far. Besides traditional approaches such as stratification studies of sparsely labeled of RGCs (whether or not combined with ChAT labeling of the inner plexiform layer strata), in vitro electrophysiological recordings and immunostainings with validated markers for specific RGC subtypes, the advent of spatial and single-cell RNA-sequencing technologies may allow identification of RGC subtypes with low versus high resistance to cell death. A recent single-cell RNA-sequencing study by Tran et al. for the first time shed light on this, and identified gene expression programs associated with differential resilience^56^. It would be informative to correlate their findings with data on the morphological changes of RGCs in response to optic nerve injury, including changes in cell and dendritic field size.

Finally, this study provides evidence to suggest that sampling of RGC population morphology and spatial distribution may have a potential role in the early detection of severe neurodegenerative disease. As assessment of the entire RGC population in humans in vivo is currently not possible, the purpose of this part of the study was to show that considering only subsets of the retinal population, such as those observed using emerging AO-cSLO retinal imaging technologies, can be used to provide a promising reliable diagnostic indicators of disease.

## Supplementary information

Supplementary Information 1.

## Data Availability

The datasets generated during and/or analysed during the current study are available from the corresponding author on reasonable request.
