# Supplementary material for: Dynamic changes in cell size and corresponding cell fate after optic nerve injury
Source: Sci Rep. 2020 Dec 10;10:21683. doi: 10.1038/s41598-020-77760-1 (PMC7730151; doi:10.1038/s41598-020-77760-1)
Supplement: Supplementary file 1 — Supplementary Information 1. [file 41598_2020_77760_MOESM1_ESM.pdf]

SUPPLEMENTARY INFORMATION

Dynamic Changes in Cell Size and Corresponding Cell Fate After Optic Nerve Injury

Benjamin M Davis[a,b], Li Guo[a], Nivedita Ravindran[a], Ehtesham Shamsher[a], Veerle Baekelandt[d], Hannah Mitchell[e], Anil A Bharath[f,] Lies De Groef[c]*, M Francesca Cordeiro[a]

[a]Institute of Ophthalmology, University College London, EC1V 9EL London, United Kingdom.

[b] Western Eye Hospital, ICORG, Imperial College London, NW1 5QH London, United Kingdom.

[c] KU Leuven, Neural Circuit Development and Regeneration Research Group, Department of Biology, Naamsestraat 61, 3000 Leuven, Belgium.

[d] KU Leuven, Laboratory for Neurobiology and Gene Therapy, Department of Neurosciences, Kapucijnenvoer 33, 3000 Leuven, Belgium.

[e] School of Mathematics and Physics, Queens University Belfast, Belfast, Ireland.

[f] Department of Bioengineering, South Kensington Campus, Imperial College London.

* shared senior authors

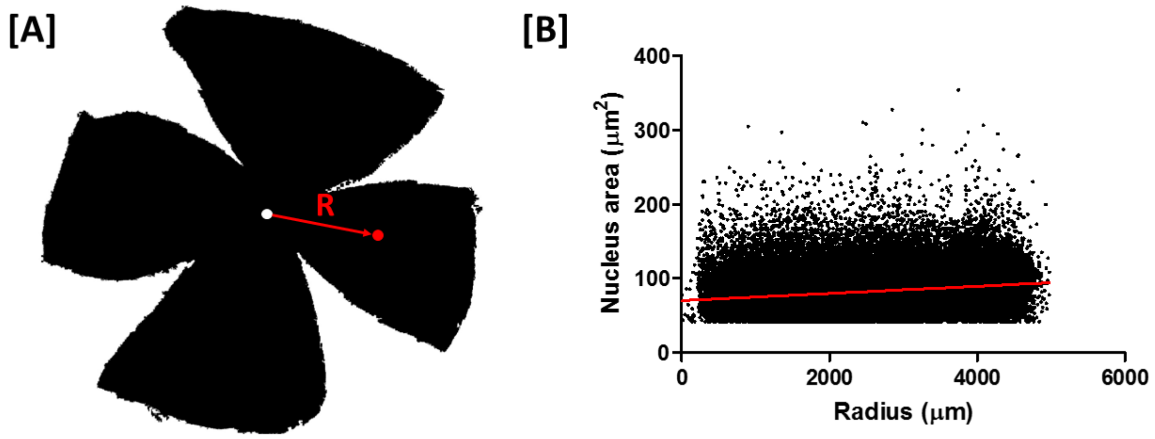

<u>Suppl Figure 1</u> **Relationship between RGC size and distance from the optic nerve head.** There is a positive correlation between RGC nucleus size and distance from the optic nerve head in the healthy retina. [A] Outline of a flatmounted retina, with the distance between the optic nerve head (white) and an RGC (red), termed the radius (R), shown. This parameter was calculated for each RGC in every retina before correlating against RGC area. [B] A significant positive correlation was observed in naïve retinas (Spearman's correlation coefficient = 0.26, $p<0.0001$).

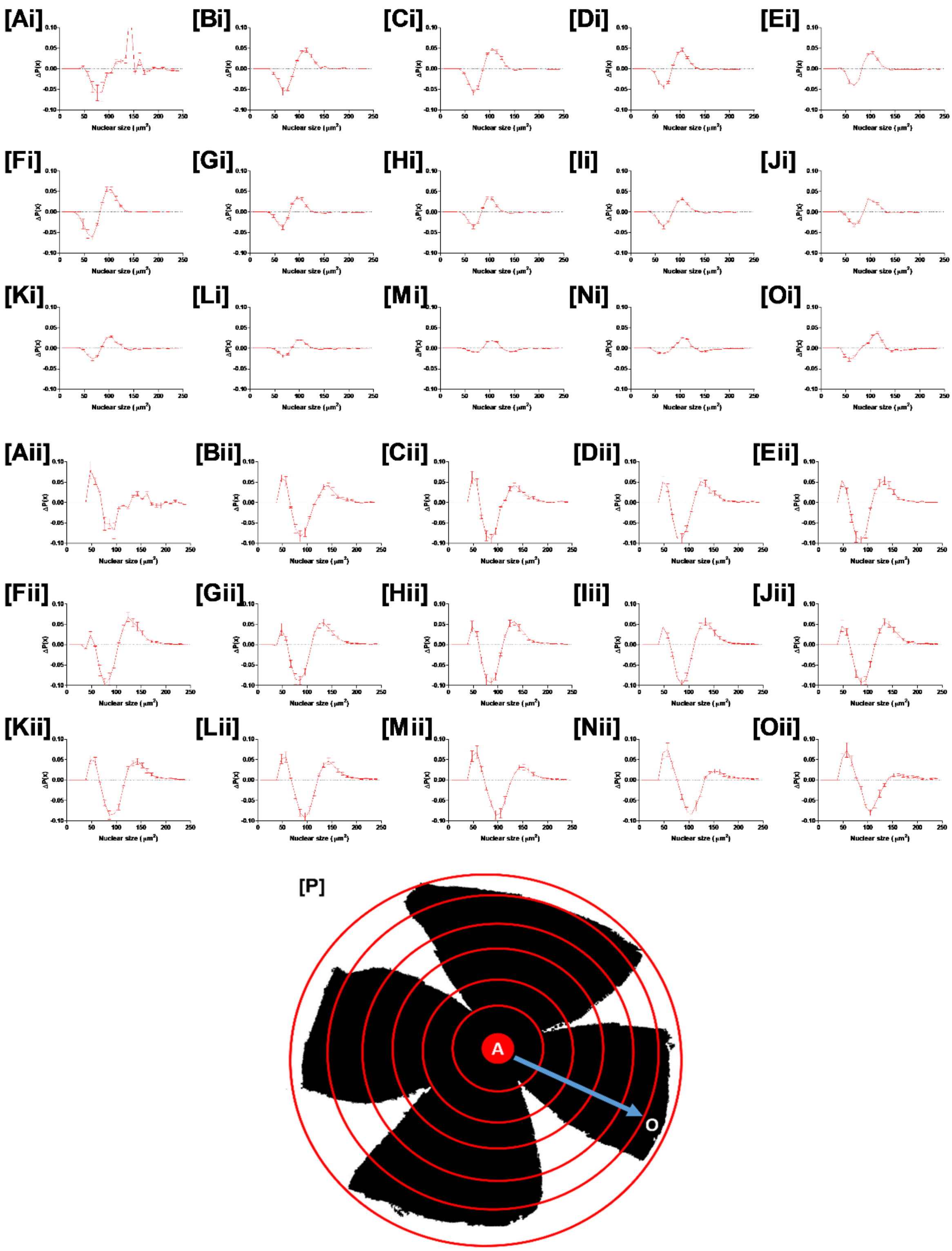

Suppl Figure 2. Investigating the spatial dependence of RGC loss relative to nucleus size and distance from the optic nerve head. [A-O] A similar pattern of RGC loss by nucleus area was observed at all distances from the ONH in both [Ai-Oi] OHT and [Aii-Oii] pONT models. This suggests that RGC position does not significantly contribute to the greater susceptibility for small RGCs to die *versus* larger cells. [P] Illustration of the retinal segmentation into non-overlapping concentric rings [A-O] centred on the ONH (not all rings shown for clarity).

RGC nuclear area (µm²)	OHT				pONT			
	0 vs 7 days	0 vs 21 days	0 vs 56 days	0 vs 84 days	0 vs 3 days	0 vs 7 days	0 vs 21 days	0 vs 56 days
**9.522**	P > 0.05	P > 0.05	P > 0.05	P > 0.05	P > 0.05	P > 0.05	P > 0.05	P > 0.05
**19.04**	P > 0.05	P > 0.05	P > 0.05	P > 0.05	P > 0.05	P > 0.05	P > 0.05	P > 0.05
**28.57**	P > 0.05	P > 0.05	P > 0.05	P > 0.05	P > 0.05	P > 0.05	P > 0.05	P > 0.05
**38.09**	P > 0.05	P > 0.05	P > 0.05	P > 0.05	P > 0.05	P > 0.05	P > 0.05	P > 0.05
**47.61**	P > 0.05	P > 0.05	P > 0.05	P > 0.05	P > 0.05	P > 0.05	P > 0.05	P > 0.05
**57.13**	P > 0.05	P > 0.05	P > 0.05	**P < 0.01**	P > 0.05	P > 0.05	**P < 0.001**	**P < 0.001**
**66.65**	P > 0.05	**P < 0.001**	**P < 0.05**	**P < 0.001**	**P < 0.001**	**P < 0.001**	**P < 0.001**	**P < 0.001**
**76.18**	**P < 0.01**	**P < 0.001**	**P < 0.001**	**P < 0.001**	**P < 0.001**	**P<0.001**	**P < 0.001**	**P < 0.001**
**85.70**	**P < 0.01**	**P < 0.001**	**P < 0.001**	**P < 0.001**	**P < 0.001**	**P<0.001**	**P < 0.001**	**P < 0.001**
**95.22**	P > 0.05	**P < 0.001**	**P < 0.001**	P > 0.05	**P < 0.001**	**P<0.001**	**P < 0.001**	**P < 0.001**
**104.7**	P > 0.05	P > 0.05	**P < 0.001**	P > 0.05	**P < 0.001**	**P<0.001**	**P < 0.001**	**P < 0.001**
**114.3**	P > 0.05	P > 0.05	P > 0.05	P > 0.05	P > 0.05	**P<0.001**	**P < 0.001**	**P < 0.001**
**123.8**	P > 0.05	P > 0.05	P > 0.05	P > 0.05	P > 0.05	P > 0.05	**P < 0.001**	**P < 0.001**
**133.3**	P > 0.05	P > 0.05	P > 0.05	P > 0.05	**P < 0.001**	P > 0.05	**P < 0.05**	P > 0.05
**142.8**	P > 0.05	P > 0.05	P > 0.05	P > 0.05	**P < 0.01**	P > 0.05	P > 0.05	P > 0.05
**152.4**	P > 0.05	P > 0.05	P > 0.05	P > 0.05	P > 0.05	P > 0.05	P > 0.05	P > 0.05
**161.9**	P > 0.05	P > 0.05	P > 0.05	P > 0.05	P > 0.05	P > 0.05	P > 0.05	P > 0.05
**171.4**	P > 0.05	P > 0.05	P > 0.05	P > 0.05	P > 0.05	P > 0.05	P > 0.05	P > 0.05
**180.9**	P > 0.05	P > 0.05	P > 0.05	P > 0.05	P > 0.05	P > 0.05	P > 0.05	P > 0.05
**190.4**	P > 0.05	P > 0.05	P > 0.05	P > 0.05	P > 0.05	P > 0.05	P > 0.05	P > 0.05
**200.0**	P > 0.05	P > 0.05	P > 0.05	P > 0.05	P > 0.05	P > 0.05	P > 0.05	P > 0.05
**209.5**	P > 0.05	P > 0.05	P > 0.05	P > 0.05	P > 0.05	P > 0.05	P > 0.05	P > 0.05
**219.0**	P > 0.05	P > 0.05	P > 0.05	P > 0.05	P > 0.05	P > 0.05	P > 0.05	P > 0.05
**228.5**	P > 0.05	P > 0.05	P > 0.05	P > 0.05	P > 0.05	P > 0.05	P > 0.05	P > 0.05
**238.1**	P > 0.05	P > 0.05	P > 0.05	P > 0.05	P > 0.05	P > 0.05	P > 0.05	P > 0.05

Suppl Table 1. Comparison of histograms of RGC density, binned by RGC nuclear size, over the natural history of OHT and pONT models. Two-way ANOVA with Bonferroni *post hoc* tests compared the RGC density to the distribution observed in naïve retina (Figure 2C & D). Regions where a significant reduction in RGC density *versus* naïve controls were observed are shown in red, whereas regions where a significant increase in RGC density versus naïve controls was observed are shown in blue.
